# Urinary tract infections in patients with renal insufficiency and dialysis – epidemiology, pathogenesis, clinical symptoms, diagnosis and treatment

**DOI:** 10.3205/id000076

**Published:** 2021-12-21

**Authors:** Jürgen E. Scherberich, Reinhard Fünfstück, Kurt G. Naber

**Affiliations:** 1 München Klinik Harlaching, Teaching Hospital of LMU Munich and KfN Munich, Germany; 2 Gesundheitszentrum Weimar, Germany; 3 Department of Urology, Technical University Munich, Germany

**Keywords:** urinary tract infection, pyuria, pyelonephritis, bacterial interstitial nephritis, chronic renal failure, end-stage kidney disease, renal replacement therapy, risk profiles, antibiotic therapy, microbial resistance

## Abstract

Epidemiological studies show an increasing number of patients worldwide suffering from chronic kidney diseases (CKD), which are associated with a risk for progression to end-stage kidney disease (ESKD). CKD patients stage 2–5, patients with regular chronic dialysis treatment (hemo- or peritoneal dialysis), and patients suffering from kidney allograft dysfunction are at high risk to develop infections, e.g. urinary tract infections (UTI) and/or sepsis (urosepsis). These groups show metabolic disturbance, chronic inflammation, and impaired immunocompetence. *Escherichia coli* is still the most common pathogen in UTI. A wide variety of other pathogens may be involved in UTI. Urological interventions, catheterization, as well as repeated courses of antibiotics contribute to an increased challenge of antimicrobial resistance. The diagnosis of UTI in CKD is based on standard clinical and laboratory criteria. Pyuria (≥10 leucocytes/µl) is more often observed in patients with oligoanuria and low bacterial colony counts. The treatment strategies for this population are based on the same principles as in patients with normal renal function. However, drugs cleared by the kidney or by dialysis membranes need dose adjustment. Antimicrobials with potential systemic toxicity and nephrotoxicity should be administered with caution.

## 1 Introduction

Chronic kidney disease (CKD) is a very common problem especially in elderly patients. Approximately 11% of the US adult population (20 million people from 1988 to 1994) are suffering from CKD at various stages. The CKD stages are defined in Table 1 (Tab. 1). The prevalence of early CKD stages (CKD stages 1 to 4; 10.8%) is more than 100 times higher than the prevalence of kidney failure (CKD stage 5; 0.1%) [1]. The prevalence of ESKD in Germany is listed in Table 2 (Tab. 2) [2].

The typical sign of kidney injury is loss of renal function, as indicated by a reduced estimated glomerular filtration rate (eGFR). CKD is defined as a clinical condition where eGFR persists abnormally (≤60 ml/min/1.73 m^2^) for more than 3 months, often combined with proteinuria. Beside morphological alterations and tissue remodelling within the kidney (interstitial fibrosis, microvascular rarification and calcification, nephron loss), various other serious alterations are involved in these processes, e.g. alterations of calcium-phosphate and vitamin-D metabolism (hyperparathyreoidism, vascular calcification), electrolyte and water imbalance (impaired volume shift, hyperkalemia), metabolic acidosis, microinflammation, dysregulation and instability of blood pressure (arterial hypertension and hypotension) or endothelial dysfunction, increased risk of cardiovascular events, mediasclerosis, stroke, maldigestion, sarkopenia, frailty, and immunodysfunction.

Clinical, biochemical, and immunological risk factors that contribute to UTI in chronic renal failure:

- Congenital abnormalties of kidney and urinary tract (CAKUT)
- Other malformations
- Urological (urogenital) surgery & instrumenting and devices
- Frequent admission to hospital, e.g. ICU
- Presence of indwelling catheters
- Nephrostoma
- Impaired glucose tolerance, Diabetes mellitus
- Chronic prostatitis; recto-ano-genital syndrome
- Nephrolithiasis, renal tubular acidosis, nephrocalcinosis
- Urolithiasis (ureter, bladder, calculi)
- Autosomal dominant polycystic kidney disease (ADPKD)
- Diverticulae of the bladder, urethra
- Inborn metabolic syndromes (cystinosis, oxalosis, cystinuria)
- Medullary cystic kidney disease
- Reflux (pelvirenal, ureter, vesicoureteral)
- Female pelvic floor dysfunction
- Neurological disorders, bladder incontinence, fecal incontinence
- Senile brain syndrome; low fluid intake, malnutrition
- Comorbidities: lymphoma, myloma, cancer, etc.
- Urethral stenoses; endoluminal valves
- Cancer of the urothel
- Interstitial nephritis
- Phagocytosis capacity, recruitment of immunocompetent cells
- Hypoproteinaemia, anaemia, uremia
- Innate immunity (monocytes, lymphocytes, dendritic cells)
- Immunoglobulin subclass deficiency
- Blood group B, AB, and non-secretor of blood group component
- Complement activity, -activation (aquired, genetic alterations)
- Mutations of *UMOD* gene (low to very low biosynthesis of Uromodulin)
- RANTES polymorphism (deficiency)
- Immunosuppressive and cytotoxic drugs
- Drugs/toxins altering the urothelial mucosal barrier
- Disturbed microbioma of the intestine and the urinary tract
- Parenteral iron supplementation (peaking levels), iron overload
- Sodium-glucose-cotransporter-2 inhibitors (SGLT2 inhibitors)

Only little data exist on how many of these patients are suffering from urinary tract infections (UTI). The incidence of infections associated with CKD is less than 1 per 5,000 people per year [3], [4], [5]. Nevertheless, frequent UTI episodes increase the risk of developing ESKD [5]. In infants and young children, the incidence is higher than in adults, but still moderate at about 1% [6]. Clinical risk factors and comorbidities other than UTI as mentioned above are suspected to be of more relevance for developing ESKD. In CKD patients, a variety of predisposing factors responsible for developing UTI includes gender, age, genetic disposition, diabetes mellitus, obstructive nephropathy, arteriolosclerosis (microvascular calcification, ischaemic nephropathy), nephrolithiasis, cast-nephropathy, immunodeficiency syndromes, and immunosuppressive therapy [3], [7].

## 2 Material and methods

A literature search was performed in various data bases, e.g. Medline, the Cochrane Database, as well as Embase; and publications of Karger, Elsevier, Klywer, Springer, Thieme, Urban & Fischer, and Dustri were studied. The following keywords were used: urinary tract infection/UTI, renal insufficiency, kidney insufficiency, end-stage renal disease/end-stage kidney disease, diseases of the kidneys, dialysis (hemo-/peritonealdialysis), microbiome, microorganisms of the urinary tract, obstruction, antibiotic treatment, prophylaxis in recurrent UTI, evaluation of renal function, and bacteriuria. Only publications with published abstracts were evaluated.

## 3 Risks of UTI and comorbidities

Apart from the impaired immune defence associated with CKD, the risk for developing UTI also depends on the underlying kidney disease. High-risk groups are patients suffering from diabetic nephropathy, nephrotic syndrome, and hypoproteinaemia, patients with analgesic nephropathy, autosomal dominant polycystic kidney disease, Randall plaques (suburothelial interstitial Ca/P nanoparticles), and renal stone formers. Among them, one may also find those with congenital errors of the metabolism: cystinosis, oxalosis, chloride channel mutations, Fabry’s disease, Dent’s disease, Bartter syndromes (e.g. Bartter type V), renal tubular acidosis, idiopathic hypercalciuria, hypocitraturia, familiar hypomagnesiaemia, nephrolithiasis, genetic defects of the calcium-sensing receptor, etc. Obstructive nephropathy associated with an increased risk for UTI may arise from papillary necrosis and/or exfoliation of tissue debris into the tubular lumen, most commonly in ischaemia as well as diabetic and analgesic nephropathy. A variety of primary kidney diseases (e.g. minimal change glomerulonephritis (GN), membranous GN, focal sclerosing glomerulopathy) are treated with immunosuppressive agents. Early diagnosis of UTI may be missed easily in those patients with no or only minor clinical symptoms. In a similar manner, CKD patients suffering from systemic vasculitis, autoimmune disease, and renal allograft dysfunction should be closely monitored for UTI. Typical signs of an infection are often completely missing in these cases.

In patients with mild and moderate CKD (stages of CKD see Table 1 (Tab. 1)), and with an anatomically normal urinary tract, lower UTI (cystitis) can be classified as ‘uncomplicated’. However, any severe infection – such as an acute episode of pyelonephritis or an uroseptic syndrome, UTI in patients with ESKD undergoing dialysis, with urinary tract abnormalities, polycystic kidney disease, urolithiasis, or unstable metabolism (diabetes mellitus) – should be classified as ‘complicated’. Under these conditions, preexisting chronic renal failure may progress rapidly (‘acute to chronic’).

## 4 Pathogenetical aspects

### 4.1 Uropathogens

There are only few studies that evaluate the uropathogens involved in patients with UTI and renal insufficiency, or in patients undergoing dialysis. *Escherichia coli* strains still remain the most common infecting organism. In addition, a wide variety of other bacteriae, such as *Proteus mirabilis*, *Pseudomonas aeruginosa*, *Enterobacter* spp., *Staphylococci*, and *Enterococci* were detected to cause UTI in these patients [8], [9], [10], [11].

Nosocomial exposure, (semi-)invasive urological interventions, and catheterizations in combination with repeated courses of antimicrobial therapy increase the risk of overgrowth of organisms with augmented antimicrobial resistance. Due to various comorbidities, CKD patients and hemodialysis (HD) patients are at high risk of being admitted to a hospital and to an intensive care unit (ICU) more frequently than non-CKD patients. Because of the immunocompromised status in CKD, problems arise from vascular access complications, chronic blood loss, frequent treatments regarding the extracorporeal circuit, alterations of the intestinal microbiome, etc. All these factors contribute to higher rates of hospitalization. Focusing on patients admitted to the ICU, the incidence of contracting a severe nosocomial infection and of being colonized with multidrug-resistant (MDR) microbes is usually increased. MDR pathogens most frequently include methicillin-resistant *Staphylococcus aureus* (MRSA), vancomycin-resistant enterococci (VRE), *Acinetobacter* spp., *Klebsiella* spp., *Morganella morganii*, and *E. coli*, often resistant also to carbapenemes. Unfortunately, there are no detailed studies characterizing the properties of virulence factors in uropathogens. In selected febrile patients, also rare uropathogens, e.g. *Leptospira *bacteria, should be excluded [12]. Leptospirosis, as a kidney-prone infection, may involve patients in both acute and chronic infections (acute renal failure, chronic tubulointerstitial nephritis, interstitial fibrosis). The problem of CKD and UTI in CKD patients is also a problem of people living in low-income regions where the incidence of diseases is higher compared to those living in wealthy nations.

Recent research indicates that the urinary tract (UT) is hosting specific and complex bacterial communities, the so-called ‘urinary microbiome’, regularly present in healthy persons. It is speculated that it might have implications for maintaining urinary health [13]. This suggests that UTI also result from an ‘imbalance’ in the UT microbiome. Future studies will demonstrate whether or not the urinary microbiome reveals protective or pathogenic properties, and if antibiotic therapy impacts its microbial pattern [14], [15]. The specific role of the microbiome in patients with CKD also needs to be better evaluated and considered [16], [17], [18].

Infections caused by hantaviruses, especially UTI, are rare, but the diseases can be severe. The infection is also described as ‘nephropathia epidemica’. Hantaviruses are RNA viruses; after infection, the incubation time is in general 3 weeks, in rarer cases 5 to 60 days [19], [20]. The central phenomena behind the pathogenesis are increased vascular permeability and acute thrombocytopenia. The pathogenesis is likely to be a complex multifactorial process that includes contributions from immune responses, platelet dysfunction, and the deregulation of endothelial cell barrier functions. A genetic predisposition related to HLA type also seems to be important for the severity of the disease [21].The disease is characterized by elevated temperature, shivering fits, and oliguria, as well as functional and morphological kidney alterations. Sometimes, proteinuria and hematuria without bacteriuria are observed. Pathological findings correlate with acute interstitial nephrits combined with deteriorations of tubular structures. As there is no effective treatment or vaccine approved for use in the USA and Europe, public awareness and precautionary measures are the only ways to minimize the risk of hantavirus disease [19], [21].

### 4.2 Nanobacteria

Another unsolved issue is the urinary presence of ultra-small bacteria, so-called ‘nanobacteria’. These are about 200 nm in diameter, exhibit densly packed spirals and few ribosomes; some show hairlike appendages of the cell surface. Hjelle et al. [22] found nanobacteria or their antigens in polycystic kidney disease. Some cyst fluids were also positive for LPS antigens from *E. coli*, *Bacteroides fragilis* and/or *Chlamydia*, and *Bartonella henselae*.

### 4.3 Host defence in CKD and ESKD

Numerous data demonstrate that the immunological profile of patients with CKD or ESKD is more or less compromised with regard to their humoral and cellular adaptive and innate immunity [23], [24], [25], [26], [27], [28], [29], [30], [31], [32], [33], [34].

CKD and HD patients show elevated endotoxin serum levels and a chronic microinflammatory status, mainly due to an increased translocation of bacteria and microbial material from the intestine. In addition, endotoxins from the dialysate fluid may enter the blood compartment and contribute to chronic inflammation and a risk for infectious diseases [32], [35], [36].

Even under immunosuppression, patients with a renal transplant disclose an increase of proinflammatory blood monocytes [33]. In patients who received a renal allograft, the amount of UTI episodes showed an imbalance of all IgG subclasses, where particularly lower serum levels of the IgG4 subclass were predictive [30]. CKD patients reveal a variety of cellular defects, which explains the increased risk for infections [7], [11]. Fibroblast growth factor 23 (FGF23) is elevated in CKD; it disturbs the immune defence by inhibiting chemokine-activated leucocytes to adhere through integrins to endothelia, and blocks the transendothelial migration of neutrophil leucocytes [37]. Elderly CKD patients (eGFR 15–44 ml/min/1.73 qm) show high hospitalization rates due to urogenital infections (+180% compared to non-CKD patients) and urosepsis, complications which are associated with an extremely high (100-fold) mortality in HD patients [38], [39], [40], [41], [42], [43], [44], [45].

The importance of toll-like receptors (TLR) (pattern recognition receptors) and the role of defensins in the pathogenesis of UTI, especially TLR2 and TLR4, and alterations in the expression of TLR in CKD patients were repeatedly confirmed [32], [46], [47]. The chronic inflammatory status of CKD parallels a decrease in the cellular expression of human leukocyte antigen – DR isotype (HLA-DR), TLR2 as well as TLR4, and impairs immune defence. In addition, the activity of the hexose-monophosphate shunt pathway, responsible for maintaining an effective phagocytosis by leukocytes, is disturbed [7], [48]. Phagocytes not only kill and eliminate bacteria, they also produce cytokines and activate other components of the immune system, among them lymphocyte and monocyte proinflammatory CD14+ and CD16++ monocyte subpopulations. Alteration of granulocyte function, impaired migration, chemotaxis, degranulation, and blockade of glucose uptake potentially promote adhesion and colonisation of host target cells, usually followed by invasion after passing the mucosal barrier of the urogenital tract [11]. It is unknown to what extent there may be a shift of virulence of uropathogens in CKD patients, and how these potential alterations are influenced by an imbalance of endogenous defensins [27], [49], [50], [51]. A small subgroup of CKD and HD patients may present congenital abnormalities of the kidney and urinary tract (CAKUT), known to trigger and promote UTI [52].

In selected cases, we identified patients with cancer of the urothel (involving either the bladder, ureter, or pyelon), where symptoms of recurrent episodes of UTI preceeded the final diagnosis of the tumor. Cancer of the urothel is more often detected in CKD and HD patients, especially in those with so-called analgesic nephropathy. Differential diagnosis may include (abacterial) interstitial nephritis, IgG4-related interstitial nephritis, and autosomal tubulointerstitial kidney disease due to mutations of the *UMOD* gene [53], [54].

Immunological factors contributing to UTI are summarized in Table 3 (Tab. 3).

### 4.4. Uromodulin (Tamm-Horsfall-Uromucoid) in CKD patients

Uromodulin, a 85–95 kDa glycoprotein, exclusively synthesized by cells of the thick ascending limb of Henle’s loop, is the major urinary inhibitor of renal stone formation and acts as a potent antimicrobial kidney-specific defensin [55], [56]. Distinct epitopes of uromodulin filaments act as multivalent ligands for bacterial type-1 pili, associate with uropathogens, mediate microbial aggregation, prevent epithelial adherence, and facilitate bacterial clearance [57]. Uromodulin is not only secreted into urine (approx. 70–100 mg/day) by apical shedding from the luminal plasma-membrane, but is also transferred (as a very stable antigen) into the blood compartment by a contraluminal, basolateral cellular pathway [34]. It is very important to notice that the uromodulin concentration in urine as well as in serum decreases progressively with the stage of CKD [34], [58].

Even at very early stages of CKD, the biosynthesis of uromodulin is significantly altered, and the capacity to neutralize bacterial components is impaired. Study participants with higher uromodulin urine levels (>39 µg/ml) had lower risk for UTI compared to those in the lowest quartile (<17 µg/ml), supporting the common view of a major protective role of uromodulin against uropathogens [59]. Since patients with ESKD and those under long-lasting renal replacement therapy disclose no or only borderline low serum and – in case of residual urine output – very low urine uromodulin, these patients are at higher risk for UTI and urosepsis due to the lack of the defensin. Inheridate progressive kidney diseases, which are related to mutations of the *UMOD* gene, reveal low to very low uromodulin concentrations even before the decline in eGFR, progressive renal papillary calcification, and urolithiasis, and are at risk to develop UTI and ESKD. Furthermore, low uromodulin levels are associated with a worse metabolic profile, higher prevalence of diabetes mellitus, arterial hypertension, heart failure, and overall mortality [60]. In addition, low serum uromodulin is associated with higher concentrations of various biomarkers of inflammation in the blood such as hsCRP, TNFα, IL18, sICAM-1, MPO, where men had higher IL6 levels compared to women [61].

Selected natural factors which are important for defence and are involved in overcoming UTI are summarized in Table 4 (Tab. 4).

## 5 Diagnostic procedures

The diagnosis of UTI is mainly based on a typical symptomatology and few robust routine laboratory methods: presence of urinary viable bacteria and increased leucocytes (pyuria), or surrogate parameters such as leucocyte esterase and positive nitrite reaction. For patients with CKD, an overview of markers and recommendations for routine and advanced diagnosis of UTI are summarized in Table 5 (Tab. 5).

The criteria for diagnosing UTI in patients with renal insufficiency are similar to those used in patients with normal renal function. Some clinical characteristics should be addressed. However, in febrile patients, where renal involvement cannot be excluded (pyelitis, pyelonephritis), blood and urine analysis as well as microbiological cultures should be performed first.

In patients with ESKD treated by HD and suspicious for an UTI (elevated CRP), blood cultures can easily be saved from extracorporeal circuit lines with the highest sensitivity and specificity compared to cultures taken by venepuncture [49]. However, in this high-risk group, despite elevated CRP, leucocytosis may not be prominent. It is a diagnostic pitfall that leucocytes of HD patients may just reach upper normal levels of healthy controls or are borderline elevated only.

Some clinical symptoms of UTI may interfere with symptoms associated with kidney-related diseases, e.g. fatigue, muscle/back/joint pain, fever, erythema, loss of appetite, and weight gain. In addition, in patients with cystitis or pyelonephritis, uncharacteristic symptoms of UTI can be misinterpreted as complications of a uremic neuropathy. Furthermore, dysuria, alguria, pollakisuria, nycturia, low-grade proteinuria, and hematuria may also be present in abacterial interstitial cystitis, which might be misinterpreted as UTI, but contradicts antibiotic treatment [53].

Pyuria is defined by a white blood cell count of more than 10 cells/µl and is also of diagnostic power in UTI in CKD and HD patients. However, 31 to 53% of dialysis patients present pyuria, but do not suffer from UTI [62], [63] (evidence: level 3). The urinary leucocyte count is broadly inversely related to the urine volume [62], [63], [64] (evidence: level 2a). This may be due to increased concentrations of a constant number of leucocytes secreted from the urothelial surfaces and the kidney itself. Low urine volume in oligoanuric patients, not uncommon in ESKD and usually present in patients on maintenance hemodialysis, effects a disturbed and abnormal ‘concentration’ of leucocytes and cell debris.

The increasing prevalence of multiresistant bacteria necessitates a rapid identification of uropathogens including their pattern of antibiotic resistance, e.g. MALDI-TOF-MS identification from blood culture fluid [65].

Diluted urine in polyuric patients with CKD may reduce the bacterial counts and reveal a negative nitrite reaction on the dipstick test, thus presenting misleading data. In patients with symptoms suggestive of UTI, one should not rely on dipstick testing if urinary culture is recommended. Although ‘significant’ bacteriuria is usually defined as colony forming units (cfu) of ≥10^5^/ml, lower-count bacteriuria (<10^5^ cfu/ml) may also be pathologically relevant, particularly in cases where a single microbe is cultured and leucocytes (granulocytes, lymphocytes) are present in high numbers.

A minor part of CKD patients with transient ‘significant’ bacteriuria may not suffer from ‘true’ invasive UTI, whereas others exhibiting lower bacterial counts with pyuria, fever, elevated C-reactive protein, leucocytosis may represent ‘active’ tissue-invasive bacterial interstitial nephritis [64]. Therefore, to determine the clinical significance of specific levels of bacteriuria in patients with decreased renal function and in patients undergoing dialysis treatment, there should not be an exclusive focus on fixed numbers, but clinical aspects should be considered primarily.

The risk that pathogens cross the mucosal urothelial barrier depends on the host defence mechanisms, which may be altered in patients with renal failure [11].

Apart from bacterial uropathogens, interstitial nephritis may involve nephrotropic viruses mimicking pyelonephritis, but lacking positive urine cultures and mass pyuria. Kidney pathohistology typically reveals granulomatous interstitial nephritis including epithelial necrosis of tubuli, associated with intranuclear inclusions, interstitial inflammation, giant cells, and epithelioid macrophages. Human herpes virus 6 (HHV6) involves tubular epithelial cells of the distal nephron and lymphocytic interstitial infiltrations. Interstitial nephritis due to polyoma virus infection shows positive SV-40 large-T antigen staining of nuclei of infected tubular cells, tubular atrophy, and urinary excretion of so-called ‘decoy cells’. In addition, infections with adenovirus (e.g. type 11) are usually accompanied by hemorrhagic cystitis. In all cases, elevated urinary α-1-microglobulin (>15 mg/g creatinine) indicates decreased epithelial reuptake of the biomarker due to tubular epithelial injury.

Patients with inflammatory (autoimmune) kidney diseases (chronic immunosuppression), diabetes mellitus, patients after urological procedures, and patients with indwelling urinary catheter are prone to develop funguria (candiduria), characteristically after inappropriate antibiotic therapy. Asymptomatic funguria may trigger or be caused by serious and potentially life-threatening candiaemia. In cases with ‘sterile’ pyuria, often associated with microhematuria, and ‘negative’ urine cultures, dysuria, fever of unknown origin, abdominal pain, and (micro)calcifications of the kidneys also involving the ureters, urogenital tuberculosis should be ruled out. In the potential risk group of migrants, micro/macrohematuria, dysuria, alguria, fever, and abdominal pain appear suspicious for urogenital schistosomiasis (prevalence in Nigeria 26–40%). Praziquantel is the drug of choice; and clinical reports address that in CKD stage 1–5, no dose adaptation is necessary. Furthermore, migrants also have a significant risk of carrying multidrug-resistant organisms (MDRO), including multidrug-resistant Gram-negative organisms (MDRGN) and methicillin-resistant *Staphylococcus aureus* (MRSA) [66].

Intravenous urography has mainly been superceded by high resolution ultrasound, ultrathin section (spiral) computed tomography (CT), and magnetic resonance imaging (MRI). High-end ultrasound investigation including contrast media is recommended as a first-line diagnostic tool in CKD and HD patients. If modern X-ray contrast media (CM) (nonionic monomers & dimers) are necessary, a study has shown that the incidence of acute kidney injury, dialysis, and death does not differ between the CM group and the control group [67]. On the contrary, conventional i.v. contrast media are relatively contraindicated in patients with progressive decline in kidney function (eGFR), or should be administered under extreme care and supervision [68].

## 6 Treatment options

Effective treatment of UTI requires a high antimicrobial concentration in urine. This is usually achieved as many antimicrobials are excreted predominantly by glomerular as well as by tubular secretion [69]. Dosing of drugs needs to be adapted to eGFR and plasma halflives of antibiotics, which are usually prolonged in CKD.

### 6.1 Treatment strategies and dosage adjustment

The treatment strategies of UTI in renal insufficiency are mainly based on the same principals as for patients with normal renal function [70], [71]. UTI should respond rapidly, without recurrence, and no rise of resistant pathogens. Acute and chronic kidney diseases affect glomerular blood flow and filtration, tubular secretion and reabsorption, bioactivation and metabolism of antibiotics. Drug absorption, bioavailability, protein binding, distribution volume, and nonrenal clearance (metabolism) can be altered in CKD especially in hemo- and peritoneal dialysis [72], [73]. Apart from recommendations how dosing of antibiotics should be adapted in CKD and HD patients [74], drug dosing errors increase the risk of side effects and poor outcome [75], [76].

A majority of patients with early CKD show normal or increased urine volumes (KDIGO stage-1 and CKD2). Oliguria is characteristic in patients treated over years by dialysis, where the residual urine volume declines progressively until (oligo-)anuria. In this context, however, oliguria is combined with reduced ability to concentrate urine, which is also seen in polyuric acute renal failure (stage 3,4,5/K/DOQI staging system). Patients on dialysis even with residual urine output will not reach effective concentrations of antibiotics [55]. There are rare reports that in anuric patients, i.v. treatments with antibiotics (cephalosporins, aminoglycosides, carbopenems) of recurrent UTI were successful [77].

The dosage of those drugs that are preferentially cleared by the kidney needs to be adjusted according to the eGFR [74], [78] (Table 6 (Tab. 6)). The calculation of renal function is only valid in a stable situation and with a constant level of endogenous serum filtration marker. Drug dosing calculated on the basis of GFR is replaced by the CKD-EPI eGFR equation (CKD-EPI = chronic kidney diseases epidemiology collaboration).

Various antimicrobial agents are cleared by the kidney either by glomerular filtration and/or tubular secretion. In CKD, this group of antibiotics requires vigorous dosing adjustment. Nomograms and electronic calculators, easily available on the Internet, are helpful for dose adjustments of antibiotics in patients with CKD and on regular chronic dialysis treatment [74]. An initial loading dose and maintenance dosing are recommended in most routinely used antibiotics [78], [79]. Dialysis therapy requires special attention with regard to dosing and which agents are cleared by the dialysis membrane (Table 7 (Tab. 7)).

Membrane clearance of drugs depends on the techniques of extracorporal treatment (hemodialysis, hemofiltration, hemodiafiltration) and the intrinsic characteristics of the dialyzer (membrane as high cut off, low cut off membrane, their hydraulic permeability, biocompatibility profile). In a similar manner, treatment modalities in patients on peritoneal dialysis (continuous technique, tidal dialysis, nocturnal peritoneal dialysis) also influence drug levels. Furthermore, the cell viability of the peritoneal membrane governs uptake (i.p. administration of antibiotics) and clearance of i.v. antibiotics, and severely alters in cases with encapsulated peritoneal sclerosis associated with high membrane transport.

### 6.2 Choice of antibiotics

The antibiotic for treatment should be chosen according to the severity of symptoms, the susceptibility of the causative microorganisms, the level of CKD, and whether or not additional comorbid factors have to be taken into account. Substances with nephrotoxic potential, e.g. aminoglycosides, should be used with great caution. Antibiotics without cumulative effects, and a wide therapeutic range index should be preferred. Broad-spectrum cephalosporins and fluoroquinolones may be effective and are the drugs of choice in this setting [80], [81], but the potential short- and long-term side effects of chinolones should be noted [82], [83], [84]. Nitrofurantoin and TMP-SMZ should be avoided in renal failure since these drugs are not sufficiently excreted into the urine and toxic serum concentration may lead to severe peripheral neuropathy [11]. Patients with CKD are also susceptible to the nephrotoxic effects of certain drug combinations, e.g. cephalosporins in addition with furosemide or ethacrynic acid [85]. In patients with impaired renal function, polymyxin/colistin should be administered with great caution because of its high potential risk of nephrotoxicity. Both polymyxins – colistin, administered as its inefficient prodrug colistimethate, and polymyxin B, administered as the active form – are cytotoxic to renal tubular cells and are prone to cause nephrotoxicity in vivo because of the renal handling mechanisms that facilitate accumulation of these compounds in these cells [86]. The available data, however, strongly suggest significantly higher rates of acute kidney injury (AKI) in patients treated with colistimethate (CMS) compared to patients treated with polymyxin B. This finding may be due to differences in pharmacokinetics and renal handling mechanisms of colistimethate and formed colistin versus polymyxin B, and consequently the relative amount of polymyxin material delivered to tubular cells. A lower risk of AKI with polymyxin B is one of several potential advantages over CMS. The relative safety and efficacy of the two agents however require closer examination in well-designed clinical studies [86]. If, however, colistin is needed because the pathogens to be treated are resistant to other possible antibiotics, underdosing patients also needs to be avoided [87]. Since a recent publication suggested that current recommendations on the use of colistin in patients with reduced renal function are likely to be inadequate, the dosing guidelines of both the European and American regulatory agencies have recently been updated [88]. In the patients requiring intermittent hemodialysis (HD), the dosing regimen of CMS should be 1.5 million international units (MIU) twice daily on non-HD days. HD should be conducted at the end of a dosing interval, and a supplemental dose of 1.5 MIU should be administered after the HD session (i.e., a total of 4.5 MIU for HD days) [89].

Other antibiotics cause (secondary) elevation of serum creatinine by mechanisms other than direct nephrotoxicity, e.g. trimethoprim inhibits tubular secretion of creatinine [90]. Oxytetracycline has an antianabolic effect in renal failure and should be avoided; doxycycline may be used, e.g. in patients suffering from urethritis. Observations suggest an antianabolic effect caused by oxytetracycline therapy [91]. Other antimicrobials (carbapenems, cephalosporins) are not contradicted in patients with CKD stages 2–5 and in dialysis patients, although dosage adjustments adapted to the level of renal impairment and drug clearance (‘dialysance’) are mandatory [63], [74].

### 6.3 Asymptomatic bacteriuria

Asymptomatic bacteriuria, ranging from 27% to 44%, has frequently been reported in CKD patients and HD patients [38], [43], [92], [93]. In patients with progressive renal disease receiving immunosuppressive agents, asymptomatic bacteriuria should probably be treated [94], pp. 898 and 1392; [95], pp. 831-2. Few retrospective studies report on increased rates of renal transplant pyelonephritis and acute rejection episodes in patients with asymptomatic bacteriuria. In CKD and HD patients (with residual urine volume excretion) and diabetes mellitus, where asymptomatic bacteriuria is accompanied by peripheral neuropathy, leucocytosis, and elevated CRP, antibiotic treatment of bacteriuria according to resistogram is recommended. However, stable patients with asymptomatic bacteriuria should not receive antibiotics.

### 6.4 Duration of antimicrobial therapy

There are no valid published data from randomized trials determining the optimal duration of treatment of UTI in patients with CKD and in dialysis patients. It is customary to treat even uncomplicated cystitis for at least 7 days and to continue for 21 days or more, depending on clinical severity [11], [70]. However, the response to even longer courses of antibiotics in higher dosage may only be transitory. Even if the urinary concentration of the antibiotic is adequate, the underlying infection may not be eradicated, thus leading to a relapse after the end of antimicrobial treatment.

Recurrent UTI presumably occur due to bacterial regrowth from colonies of non-planktonic bacteria residing in a protected biofilm environment. Persistent microbial niches may develop and colonize deeply within damaged renal parenchymal or urothelial tissue. Furthermore, antibiotic therapy may select highly resistant intracellular, ecologically stable bacterial communities living temporarily as commensals, so-called ‘small colony variants’ (SCV).

Ultimately, the available option is surgical excision of diseased tissues. Nephrectomy is only very occasionally performed in patients with incurable relapsing destructive pyelonephritis and signs of rapidly progressive CKD. Here, the majority of patients shows resistance to any further antimicrobial treatment, and suffers from a malignant form of nephrolithiasis, heavily scarred kidneys, abscess formation, severe congenital obstruction, as well as severe recurrent UTI in polycystic kidney disease.

Importantly, recent studies have confirmed again that any infection irrespective of severity is an independent risk factor for increased adverse events in the CKD population [96].

## 7 Conclusions

There is no sufficient evidence that UTI (e.g. cystitis in patients with mild or moderate CKD without urinary tract abnormalities) or asymptomatic bacteriuria generally worsens kidney function (eGFR). Leukocyturia and inflammation markers are important signs of UTI. Recurrent episodes of UTI increase the risk of ESKD in CKD patients. Any severe infection, such as acute episodes of pyelonephritis, recurrent UTI, urosepsis, or any UTI in symptomatic patients with ESKD and on dialysis with comorbidities should be adequately treated with antibiotics according to the recommendations of ‘antimicrobial stewardship’ in order to lower the rates of bacterial resistance. Unfortunately, no prospective clinical trials are available to assess the optimal management of UTI in patients with CKD stage 4–5 and in dialysis populations, respectively.

## Abbreviations

- AKI: acute kidney injury
- CAKUT: congenital abnormalities of the kidney and urinary tract
- CKD: chronic kidney disease
- CKD-EPI: chronic kidney diseases epidemiology collaboration
- CM: contrast medium
- CMS: colistimethate
- CT: computed tomography
- eGFR: estimated glomerular filtration rate
- eGFR-EPI: estimated glomerular filtration rate – epidemiology collaboration
- ESKD: end-stage kidney disease
- FGF23: fibroblast growth factor 23
- GN: glomerulopathy, glomerulonephritis
- HD: hemodialysis
- HHV6: human herpes virus 6
- HLA-DR: human leukocyte antigen – DR isotype
- ICU: intensive care unit
- IgG: immune globulin G
- KDIGO: Kidney Disease: Improving Global Outcomes (organization)
- MDR: multidrug-resistant microbes
- MRSA: methicillin-resistant *Staphylococcus aureus*
- MRI: magnetic resonance imaging
- PCR: protein chain reaction
- PPI-Inhibitor: proton secretion inhibitor
- SCV: small colony variants
- SLE: systemic lupus erythematosus
- TLR: toll-like receptor
- UT: urinary tract
- UTI: urinary tract infection
- VRE: vancomycin-resistant enterococci

## Note

This article will also be published as a chapter of the Living Handbook “Urogenital Infections and Inflammations” [97].

## Competing interests

JS declares that he has no competing interests.

RF reports consulting fees within the last two years (2019, 2020) from Novartis Pharma GmbH and Boehringer.

KN reports personal consulting fees within the last two years (2019, 2020) from Adamed, Biomerieux, Bionorica, Eumedica, Hermes, Immunotek, Janssen, Klosterfrau, Medice, OM Pharma.

## Figures and Tables

**Table 1:**
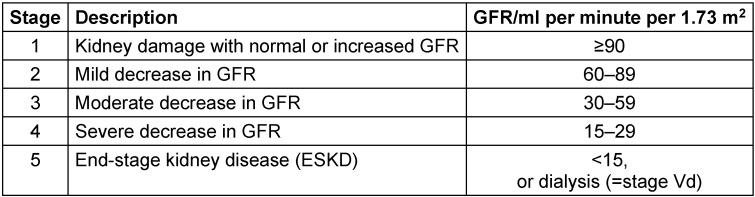
Staging system for chronic kidney disease, K/DOQI, KDIGO [1]

**Table 2:**
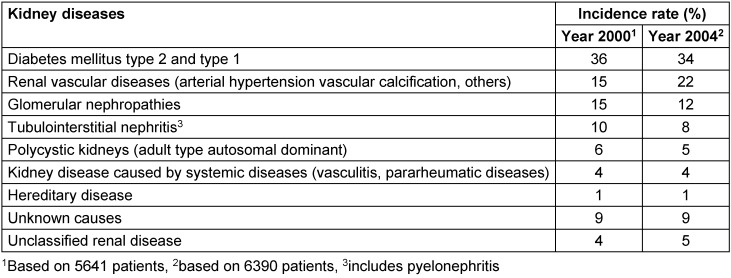
Incidence rates of various kidney diseases with potential progression to end-stage kidney disease; results of the German Dialysis Registry for the years 2000 and 2004 [2]

**Table 3:**
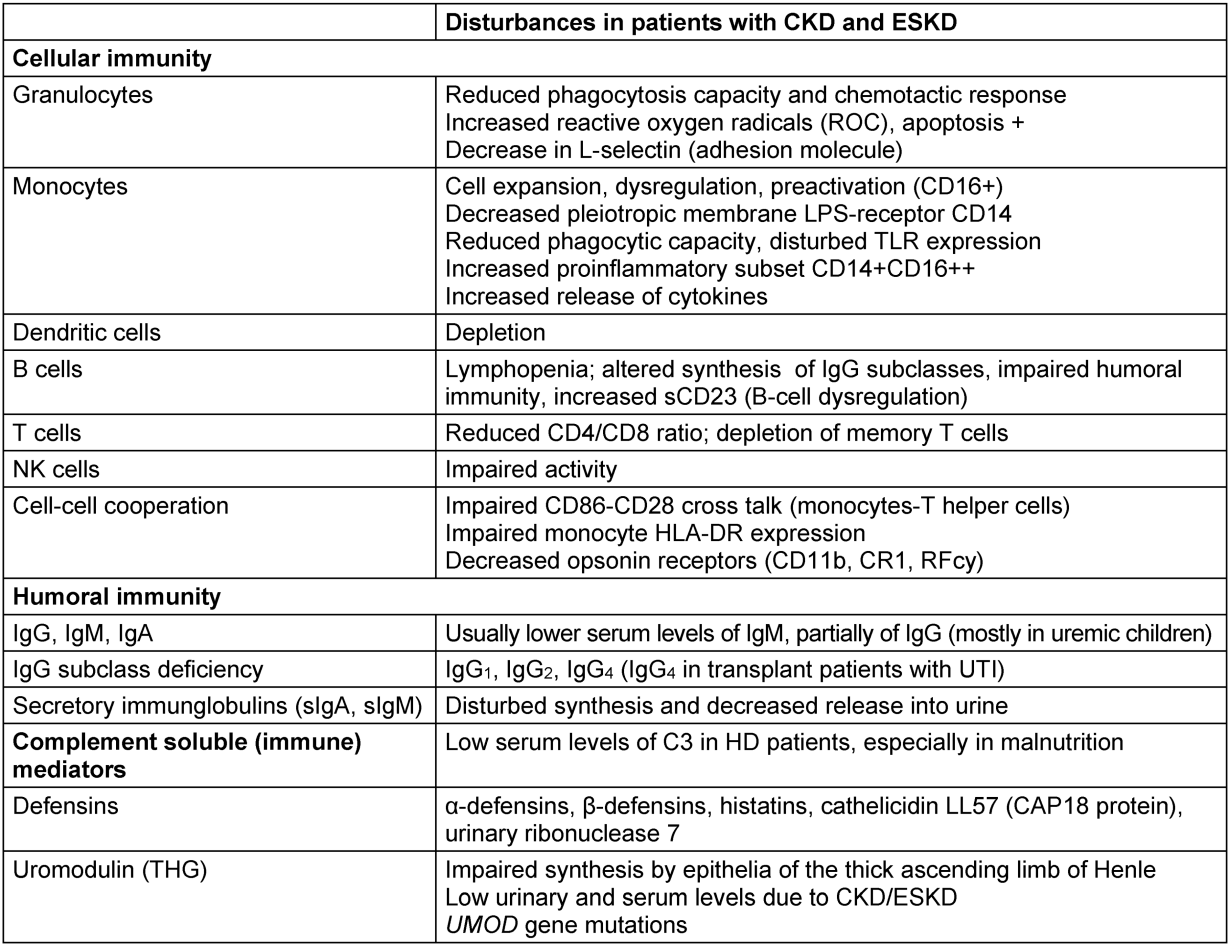
Immunological factors contributing to UTI in chronic renal failure, following kidney transplantation and in chronic dialysis patients

**Table 4:**
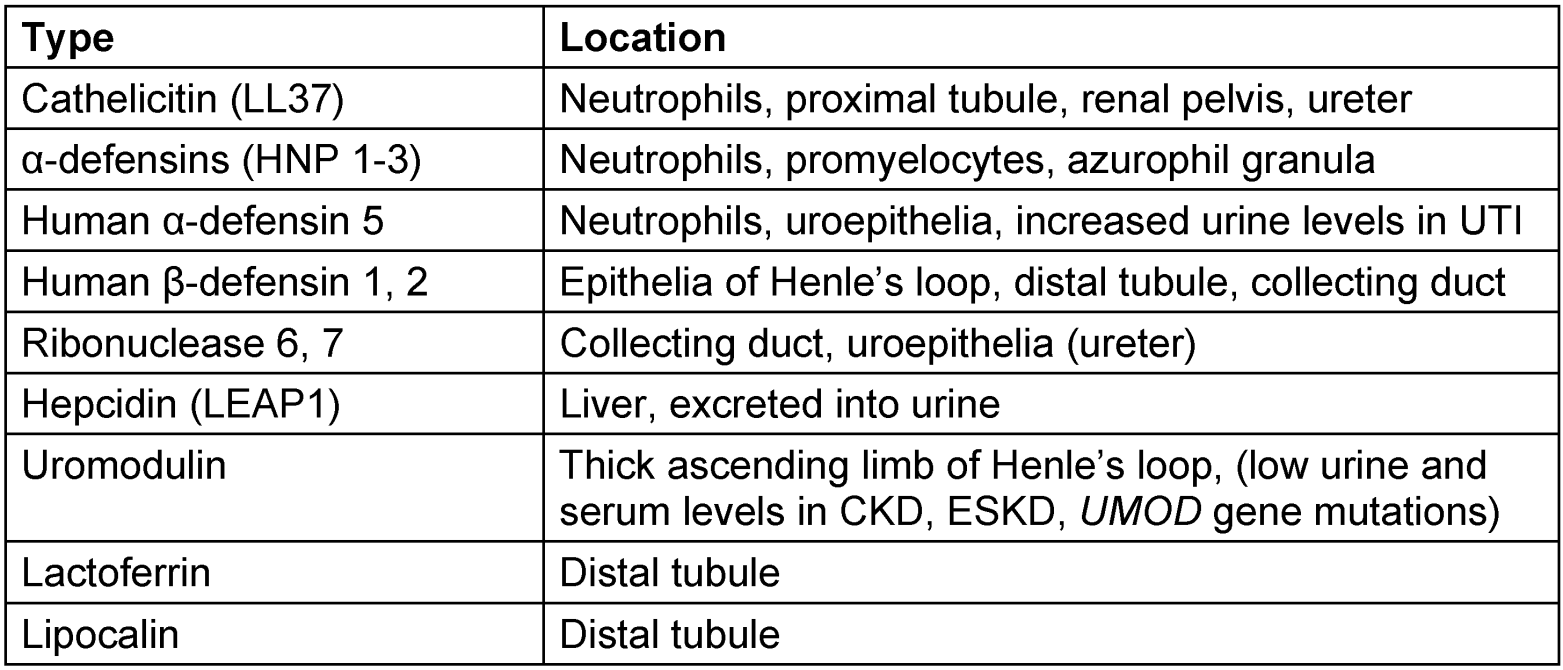
Selected natural (intrinsic) defensins involved to overcome UTI

**Table 5:**
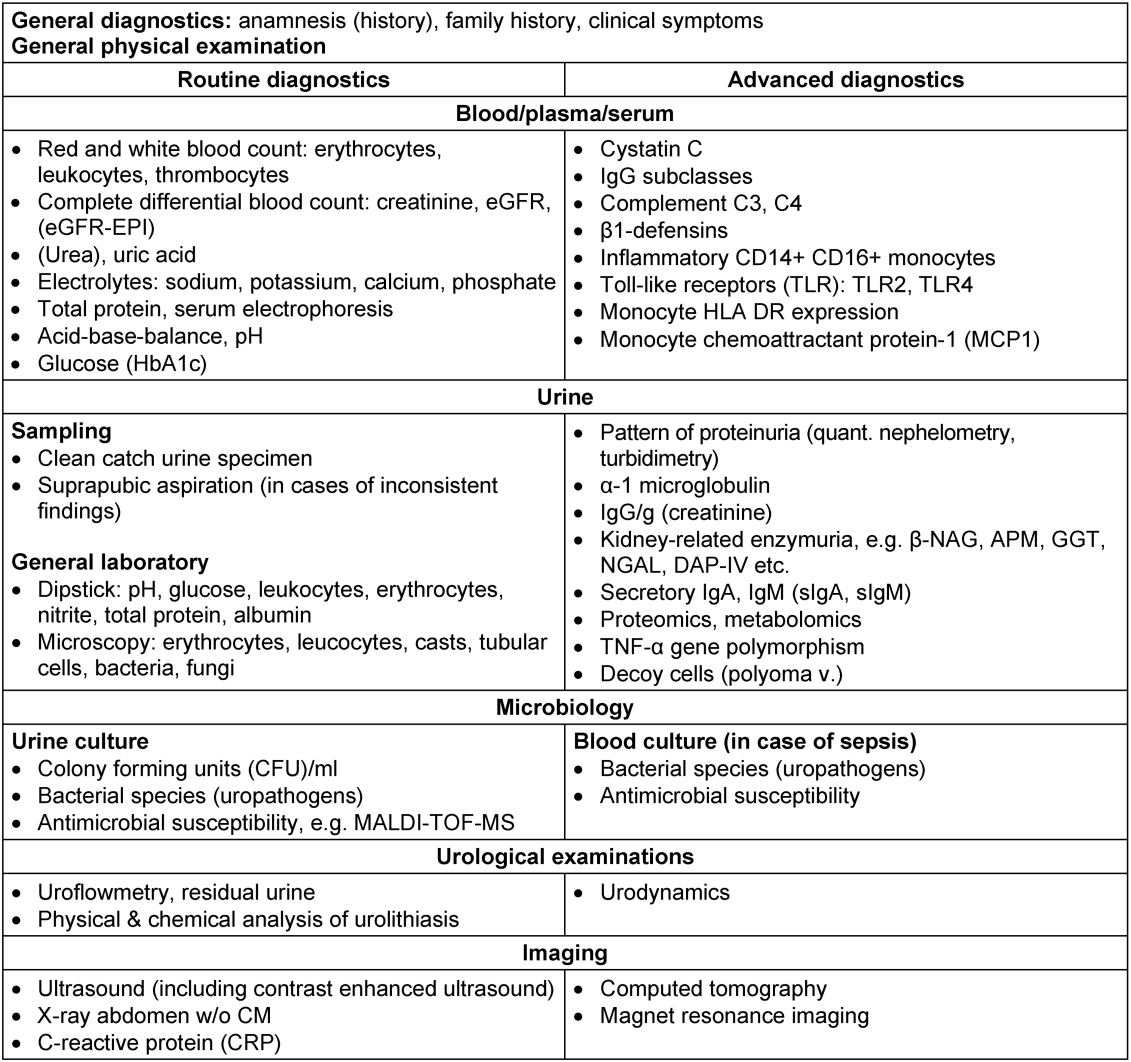
Recommendations for routine and advanced diagnostics of UTI in patients with CKD

**Table 6:**
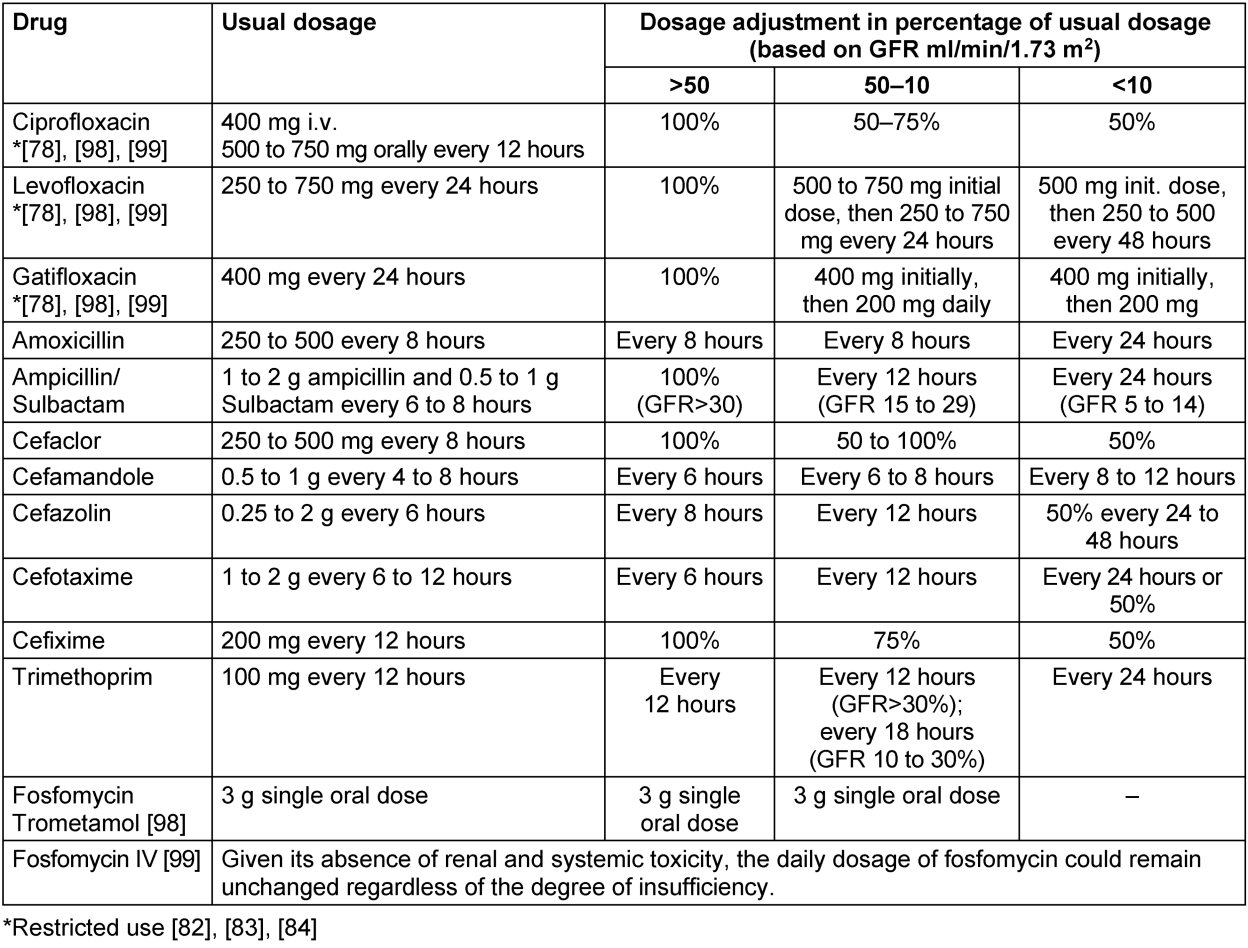
Antimicrobial agents for treatment of UTI: Dosing requirements in patients with chronic renal failure [71], [72]

**Table 7:**
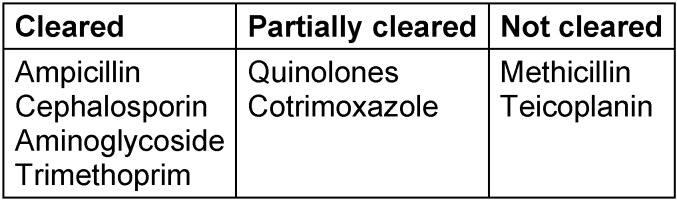
Dialysance of antimicrobial agents in patients undergoing hemodialysis treatment [79], [74]
